# Translocation of non-lytic antimicrobial peptides and bacteria penetrating peptides across the inner membrane of the bacterial envelope

**DOI:** 10.1007/s00294-021-01217-9

**Published:** 2021-11-08

**Authors:** Jakob Frimodt-Møller, Christopher Campion, Peter E. Nielsen, Anders Løbner-Olesen

**Affiliations:** 1 Department of Biology, Center for Peptide-Based Antibiotics, University of Copenhagen, Copenhagen, Denmark; 2 Department of Cellular and Molecular Medicine, Center for Peptide-Based Antibiotics, Faculty of Health and Medical Sciences, The Panum Institute, University of Copenhagen, Copenhagen, Denmark

**Keywords:** Non-lytic antimicrobial peptides, Bacteria penetrating peptides, Antimicrobial antisense agents, Bacterial membranes

## Abstract

The increase in multidrug-resistant pathogenic bacteria has become a problem worldwide. Currently there is a strong focus on the development of novel antimicrobials, including antimicrobial peptides (AMP) and antimicrobial antisense agents. While the majority of AMP have membrane activity and kill bacteria through membrane disruption, non-lytic AMP are non-membrane active, internalize and have intracellular targets. Antimicrobial antisense agents such as peptide nucleic acids (PNA) and phosphorodiamidate morpholino oligomers (PMO), show great promise as novel antibacterial agents, killing bacteria by inhibiting translation of essential target gene transcripts. However, naked PNA and PMO are unable to translocate across the cell envelope of bacteria, to reach their target in the cytosol, and are conjugated to bacteria penetrating peptides (BPP) for cytosolic delivery. Here, we discuss how non-lytic AMP and BPP-PMO/PNA conjugates translocate across the cytoplasmic membrane via receptor-mediated transport, such as the cytoplasmic membrane transporters SbmA, MdtM/YjiL, and/or YgdD, or via a less well described autonomous process.

## Introduction

Antimicrobial resistance is now a global problem, that predominantly is driven by the exaggerated use of antibiotics in human medicine and agriculture (Davies et al. 2013). One solution to ensure a viable option to treat bacterial infection in the future is the discovery and development of novel antibiotics, including antimicrobial peptides (AMP) (Shagaghi et al. 2018) and antimicrobial antisense agents (Hegarty and Stewart 2018; Pifer and Greenberg 2020). AMPs are structurally diverse and widespread peptides, many of which are present in biological protection systems of a variety of organisms including vertebrates, plants, fungi, and bacteria, e.g. as an integral part of the innate immune system in vertebrates. The majority of AMPs are membrane-active, killing bacteria by membrane disruption, while a few non-lytic AMPs act on intracellular targets. It is well established that non-membrane active AMP translocate across the bacterial cell envelope without causing damage (Boman et al. 1993; Casteels and Tempst 1994; Castle et al. 1999; Knappe et al. 2010; Podda et al. 2006) before binding to intracellular targets.

Antimicrobial antisense agents inhibit gene expression at the translational level via specific binding to sequence complementary mRNA of essential genes or to essential sites of rRNA, leading to growth cessation. Due to their biological stability, neutral charge, high binding affinity and specificity for sequence complementary RNA, synthetic nucleic acid analogs such as peptide nucleic acids (PNA) or phosphorodiamidate morpholino oligomers (PMO) are preferred as antimicrobial antisense agents (Summerton and Weller 1997; Good and Nielsen 1998a; Wesolowski et al. 2011). Antimicrobial antisense PNAs and PMOs are usually 10-12 bp long (Dryselius et al. 2003; Deere et al. 2005; Goltermann and Nielsen 2020), i.e., (MW > 3000), larger than traditional small-molecule antibiotics (MW ≤ 1000 Da), and if unmodified are unable to pass through the cell envelope (Wittung et al. 1995; Good and Nielsen 1998a, b; Good et al. 2000). To improve cytosolic delivery antimicrobial antisense agents can be covalently attached to bacteria-penetrating peptides (BPP) (Good et al. 2001; Geller et al. 2003). BPP constitute a group of peptides of natural or synthetic origin with bacterial membrane translocation capabilities, usually sharing some common characteristics such as being cationic, but also having a content of hydrophobic amino acids. Indeed, these characteristics are to a large extent shared with AMPs as well as with cell-penetrating peptides [CPP, used for cytoplasmic delivery in eukaryotic cells (Ruseska and Zimmer 2020)], and most likely reflect physical interaction with negatively charged cell membranes as a common feature involved in the mechanism of action. Consequently, a varying extent of functional overlap between AMP, BPP and CPP is often observed, e.g. in the form of cytotoxicity, but the detailed structure activity relationship differences within and between the groups is quite subtle, and far from fully understood.

The outer membrane of Gram-negative bacteria and the cell wall of Gram-positive bacteria present two different obstacles for peptide translocation. In Gram-positive bacteria AMP and BPP most likely diffuse through undefined pores in the peptidoglycan (Malanovic and Lohner 2016), while in Gram-negative bacteria they are believed to have a poorly understood autonomous uptake, likely involving interactions with lipopolysaccharide, possible outer-membrane bound proteins and/or phospholipids (Li et al. 2017). However, for most non-lytic AMP and BPP the rate-limiting step for translocation to the cytosol is the cytoplasmic membrane (Li et al. 2017). At the cytoplasmic membrane, the molecules enter either via autonomous membrane translocation and/or via a specific transporter mechanism. Here, we focus on translocation across the cytoplasmic membrane, with a special emphasis on uptake of non-lytic AMP/BPP in the Gram-negative bacterium *Escherichia coli*.

## Receptor-mediated uptake through the cytoplasmic membrane

The most well-described receptor-mediated uptake mechanism of non-lytic AMPs and BPPs is through the cytoplasmic membrane ABC transporters SbmA and BacA, belonging to the peptide uptake permease family (Saier 2000). These are found in distantly related species. For instance, SbmA is found in *Enterobacteriaceae* (Corbalan et al. 2013), and BacA in the genus *Mycobacterium* (incl. *M. tuberculosis*) (Domenech et al. 2009) and the alphaproteobacteria *Brucella abortus* and *Sinorhizobium meliloti* (Glazebrook et al. 1993; LeVier et al. 2000). In *E. coli* the 406 amino acid SbmA protein consists of eight transmembrane domains and peptide transport is driven by the electrochemical gradient (Runti et al. 2013). BacA shares high sequence similarity to SbmA (Glazebrook et al. 1993; Ichige and Walker 1997) and a *sbmA*-deficient *E. coli* mutant can be complemented by the *bacA* gene from *S. meliloti* or *M. tyberculosis*, indicating functional similarity as transporters (Ichige and Walker 1997; Domenech et al. 2009). While SbmA is a non-essential protein in laboratory settings (Pranting et al. 2008), it has been identified as a virulence factor in *E. coli* (APEC)(Li et al. 2005), and BacA is required for *Rhizobium meliloti* symbiosis establishment in plants (Glazebrook et al. 1993) and prolonged *B. abortus* and *M. tuberculosis* infections in mice (LeVier et al. 2000; Domenech et al. 2009).

**Fig. 1:**
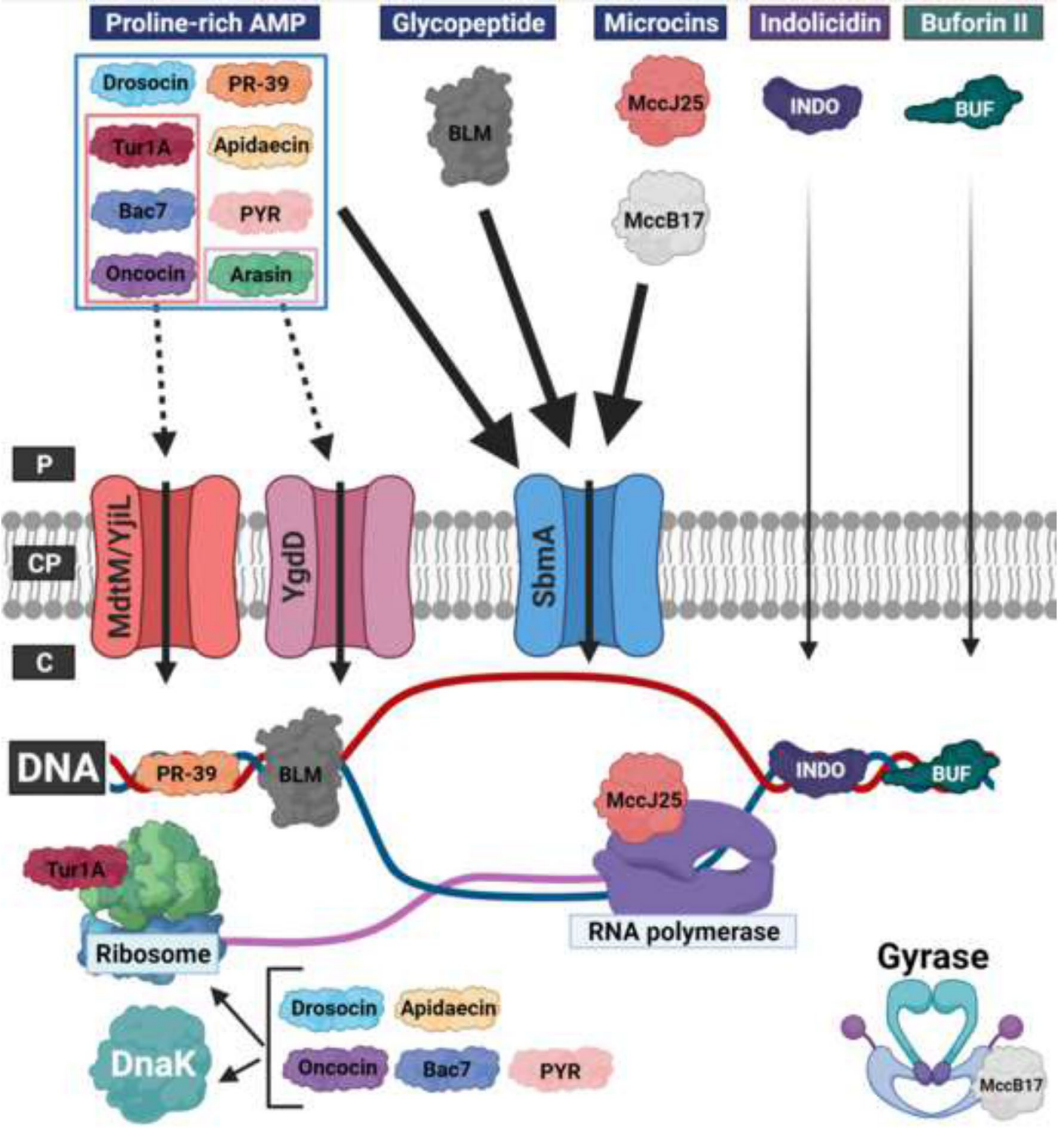
Uptake of non-lytic AMP across the cytoplasmic membrane. A schematic presentation of the periplasm (P), cytosolic membrane (CP), and cytosol (C) in *E. coli*. From the periplasm the proline-rich AMP oncocin, Bac7, arasin, apidaecin, PR-39, pyrrhocoricin (PYR), drosocin, and Tur1A translocate across the cytosolic membrane primarily through SbmA (indicated by the full line). MdtM/YjiL plays an accessory role for Tur1A, Bac7, and oncocin, while YgdD plays an accessory role for arasin. Microcins J25 (MccJ25) and B17 (MccB17) and the glycopeptide bleomycin (BLM) are translocated across the cytoplasmic membrane by SbmA. Indolicidin (INDO) and buforin II (BUF) are proposed to translocate across the outer membrane and cytoplasmic membrane by autonomous uptake. In the cytosol oncocin, Bac7, apidaecin, PYR, drosocin, and Tur1A binds to the ribosome (Krizsan et al. 2014; Gagnon et al. 2016; Mardirossian et al. 2014, 2018) and with the exception of Tur1A also DnaK [REF (Czihal et al. 2012; Knappe et al. 2011; Kragol et al. 2001; Otvos et al. 2000; Scocchi et al. 2009; Zahn et al. 2014; Zhou Y and W N Chen. 2011)] PR-39, indolicidin, buforin II and BLM to DNA (Yamamoto et al. 1984; Boman et al. 1993; Subbalakshmi and Sitaram 1998; Park et al. 2000; Kosa et al. 2004; Hsu et al. 2005), MccJ25 to the RNA polymerase, and MccB17 to gyrase (Baquero et al. 2019), which in all cases leads to growth arrest. So far, the cytosolic target of arasin has not been elucidated (Paulsen et al. 2013). See text and for details

The uptake mechanism for proline-rich AMP has in *E. coli* primarily been ascribed to transport via SbmA. These peptides include arasin (Paulsen et al. 2016), apidaecin (Mattiuzzo et al. 2007; Krizsan et al. 2015), Bac7 (Mattiuzzo et al. 2007; Runti et al. 2013; Guida et al. 2015), drosocin (Krizsan et al. 2015), oncocin (Krizsan et al. 2015), pyrrhocoricin (Narayanan et al. 2014), PR-39 (Mattiuzzo et al. 2007; Pranting et al. 2008) and Tur1A (Mardirossian et al. 2018) (Fig. 1). Analogously, BacA transports proline-rich AMPs across the cytoplasmic membrane at least in *S. meliloti* (Marlow et al. 2009; Wehmeier et al. 2010). In *E. coli* proline-rich AMPs depend on SbmA for full activity. However, additional cytoplasmic membrane transporters such as the drug/H + antiporter MdtM and the cytoplasmic membrane transporter YgdD play an accessory role to SbmA in the uptake of Tur1A/Bac7/oncocin and arasin, respectively (Krizsan et al. 2015; Paulsen et al. 2016). Thus, bacterial species, most commonly Gram-negative bacteria, containing SbmA/BacA are susceptible to proline-rich AMPs (Marlow et al. 2009). Conversely, species deficient of SbmA/BacA, such as *Pseudomonas aeruginosa*, are less susceptible to this class of AMPs (Benincasa et al. 2004; Paulsen et al. 2013; Narayanan et al. 2014; Bluhm et al. 2015; Knappe et al. 2016; Runti et al. 2017). This could also explain why Gram-positive bacteria, in general, are resistant to these. In addition, SbmA transports microcins B17 and B25 (Lavina et al. 1986; Salomon and Farias 1995) as well as the glycopeptide bleomycin (Yorgey et al. 1994; Mattiuzzo et al. 2007) in *E. coli*, while BacA transports microcins B17 and B25 in *S. meliloti* (Ichige and Walker 1997) and bleomycin in both *S. meliloti* (Ichige and Walker 1997) and *M. tuberculosis* (Domenech et al. 2009).

**Fig. 2:**
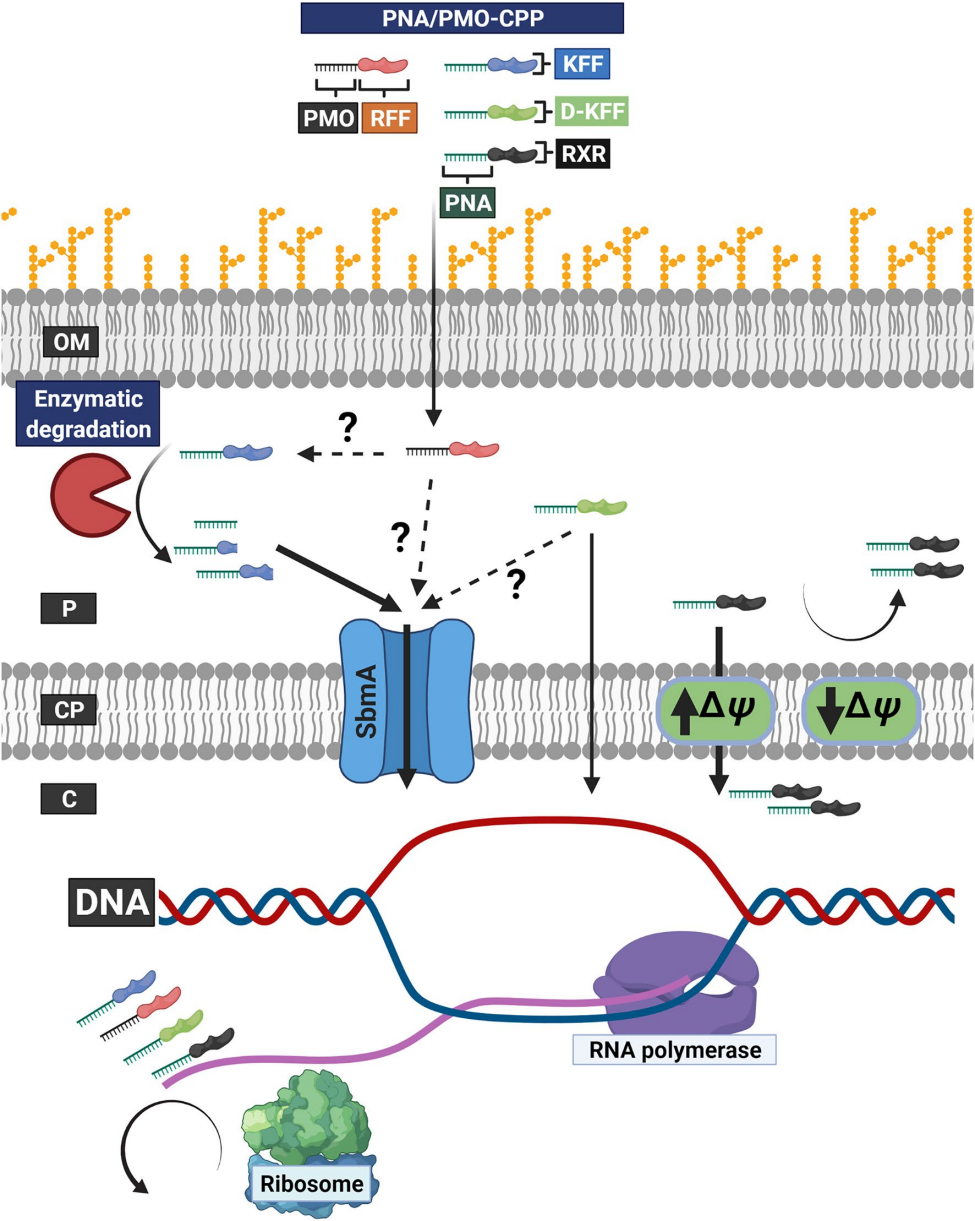
Uptake of PNA/PMO-BPP across the cell envelope. A schematic presentation of the outer membrane (OM), periplasm (P), cytosolic membrane (CP), and cytosol (C) in *E. coli*. The biostability of PNA conjugated to (KFF)_3_ K-eg1 (PNA-KFF), and possible also PMO conjugated to (RFF)_3_R-Ahx-β-alanine (indicated by a dashed line), is limited leading to truncated peptide-PNA conjugants in the periplasm. Truncated PNA-KFF conjugates and truncated/full-length PMO conjugated to (RFF)_3_R-Ahx-β-alanine are translocated across the cytoplasmic membrane by SbmA. The PNA conjugated to the D-form of (KFF)_3_ K-eg1 (PNA-D-KFF) has highly increased biostability compared to the l-form, and this peptide-PNA conjugate is believed to translocate across the cytoplasmic membrane by autonomous uptake (indicated by a full line). However, uptake through SbmA cannot be excluded (indicated by a dashed line). PNA conjugated to (R-X-R)_4_-Ahx-β-alanine (PNA-RXR) requires a high Δ*Ψ* across the cytoplasmic membrane to translocate, while a low Δ*Ψ* results in a decreased uptake. In the cytosol PNA/PMO-BPP binds to the mRNA of an essential gene, which leads to growth arrest. See text for details

PNA conjugated to the lysine/phenylalanine containing BPP (KFF)_3_ K-eg1^1^ and PMO conjugated to the arginine/phenylalanine containing BPP (RFF)_3_R-Ahx-β-alanine^2^ are transported across the cytoplasmic membrane by SbmA in *E. coli* (Puckett et al. 2012; Ghosal et al. 2013). The (KFF)_3_ K-eg1 BPP is instrumental for translocation across the outer membrane but has very limited biostability in the periplasm, resulting in peptide-truncation (Yavari et al. 2021). These truncated peptide-PNA conjugates, require SbmA-dependent uptake across the cytoplasmic membrane, while the enzymatically stable D-form of full length (KFF)_3_ K-eg1-PNA is able to cross in an SbmA-independent manner (visualized in Fig. 2) (Yavari et al. 2021). Arginine containing BPPs usually have an autonomous uptake across the cytoplasmic membrane (see below), suggesting that (RFF)_3_R-Ahx-β-alanine might face similar biostability issues in the periplasm as (KFF)_3_ K-eg1. Compared to the straightforward resistance development to (KFF)_3_ K-eg1-PNA, through loss of SbmA (Ghosal et al. 2013), resistance development to peptide-PNA translocating through an autonomous uptake in *E. coli* requires multiple mutations and are difficult to obtain(Frimodt-Møller et al. 2021). Thus, emphasizing the importance of improving carrier stability when optimizing/designing BPPs for delivery of antimicrobial peptides in bacteria.

## Autonomous uptake across the cytoplasmic membrane

Some non-lytic AMPs and BPPs do not internalize through a receptor-mediated uptake. Hypothesis derived from in silico and in vitro studies has been put forward to explain this autonomous translocation (Rothbard et al. 2004; Stanzl et al. 2013; Herce et al. 2014; Li et al. 2017), including the “self-promoted uptake” model proposed by Hancock and Chappel (Hancock and Chapple 1999). Nonetheless, it remains poorly understood how these molecules reach the cytosol in bacteria. The non-lytic AMPs indolicidin, a tryptophan/proline-rich AMP (Selsted et al. 1992), and buforin II (Park et al. 1996) enters the cytoplasm without disrupting membrane integrity (Fig. 1). Buforin II although non-proline-rich has a proline residue at position 11 (Yi et al. 1996), which plays a key role in effective translocation (Kobayashi et al. 2004).

Uptake mechanisms for arginine-rich peptides differ significantly between mammalian and bacterial cells, as endocytosis (for which no analogue exists in bacteria) is  the predominant uptake pathway in mammalian cells (Ruseska and Zimmer 2020). However, very recent results are beginning to shed new light on the SbmA independent, autonomous uptake of at least arginine-rich BPPs and AMPs. Here, an activated Cpx-response was shown to confer resistance to the (R-X-R)_4_-Ahx-β-alanine-PNA conjugate in *E. coli* (Frimodt-Møller et al. 2021). The activated Cpx-response resulted in the downregulation of respiratory genes leading to a decreased cytoplasmic membrane potential. This indicates an indirect autonomous but energy-dependent uptake mechanism, relying on a high membrane potential, for antimicrobials conjugated to such an arginine-rich BPP (Fig. 2) (Frimodt-Møller et al. 2021). In agreement, an activated Cpx-response also conferred a decreased susceptibility to aminoglycosides and another arginine-rich BPP (R_11_) conjugated to a peptide targeting the DNA sliding clamp (Frimodt-Møller et al. 2021).

These findings are of general interest for several reasons. First of all, they confirm and stress, that also for cationic peptides with autonomous, and thus transporter independent uptake, crossing the inner membrane is the main barrier in (Gram-negative) bacteria. Furthermore, the findings shed important light on the mechanism for crossing the inner membrane, by demonstrating that this requires a significant negative membrane potential to energetically drive the passage. Resistance development towards receptor translocated AMPs and BPPs are in many cases straightforward, ex. by loss of the transporter/receptor (Ghosal et al. 2013). Conversely, resistance development towards non-receptor translocated BPPs (and AMPs) are difficult to obtain and come with a high fitness cost (Frimodt-Moller et al. 2021). Thus, the uptake route is an important parameter to consider when designing/searching for novel non-lytic AMPs or BPPs, preferable pursuing a non-receptor mediated uptake.

## Footnotes

1 K refers to lysine, F refers phenylalanine, and eg1 refers to 8-amino-3,6-dioxaoctanoic acid.

2 R refers arginine and Ahx refers to 6-aminohexanoic acid.
